# Contrast-Induced Encephalopathy Following Cerebral Angiography: A Case Report and Literature Review

**DOI:** 10.7759/cureus.94497

**Published:** 2025-10-13

**Authors:** William Strickland, Shashidhar Manchegowda, Vathsala Javagal Krishnamurthy, Satya Patro

**Affiliations:** 1 Anesthesiology, University of Arkansas for Medical Sciences, Little Rock, USA; 2 Anesthesiology Critical Care, University of Arkansas for Medical Sciences, Little Rock, USA; 3 Psychiatry, Arkana Laboratories, Little Rock, USA; 4 Radiology, University of Arkansas for Medical Sciences, Little Rock, USA

**Keywords:** brain disease, cerebral angiography, contrast dye, contrast-induced encephalopathy, iodinated contrast media, neurotoxicity syndromes, stroke mimic

## Abstract

Contrast-induced encephalopathy (CIE) is a rare neurotoxic complication that occurs following administration of contrast agents. Diagnosis is often challenging due to the wide variety of clinical presentation, which can include transient neurological deficits such as vision changes, sensory deficits, paralysis, and altered mental status. It is self-limited, and symptoms usually resolve within 48-72 hours. It is important to consider contrast-induced encephalopathy in a patient that experiences neurological defects following procedures using contrast agents. This report describes a 58-year-old male who subsequently developed contrast-induced encephalopathy following a diagnostic cerebral angiogram.

## Introduction

Contrast-induced encephalopathy (CIE) is a rare neurotoxic complication following the intravenous or intra-arterial administration of an iodinated contrast agent during procedures such as CT imaging and cerebral angiography [1]. Clinical manifestations of contrast-induced encephalopathy often vary with a wide range of symptoms including visual disturbances, motor defects, sensory defects, and confusion [2]. Incidence of acute transient neurologic deficits is thought to be around 0.51% per 10,000 patients [3]. Prognosis is usually favorable, with most patients experiencing full resolution of symptoms within 48-72 hours [4].

The mechanism of contrast-induced encephalopathy remains poorly understood. Providers are widely in agreement that the topic warrants further investigation, as a recent survey demonstrated that 86.3% of providers believed that the pathophysiology was unclear [5]. It has been proposed that a key step in the pathogenesis of contrast-induced encephalopathy is likely blood-brain barrier (BBB) breakdown [6,7]. Often, BBB breakdown can be visualized on imaging findings through mass effect with edema, however, sometimes no radiographic features are visible [8].

We present a case of contrast-induced encephalopathy with no radiographic findings following diagnostic cerebral angiography.

## Case presentation

A 58-year-old man with an unremarkable medical history was admitted for scheduled diagnostic cerebral angiography to evaluate a left supraclinoid internal carotid artery aneurysm. This aneurysm had been discovered incidentally two weeks earlier during CT angiography of the head and neck, performed when he presented to the Emergency Department following a slip and fall during which he hit his head and briefly lost consciousness. He had been discharged home in stable condition with instructions to return for follow-up diagnostic imaging.

**Figure 1:**
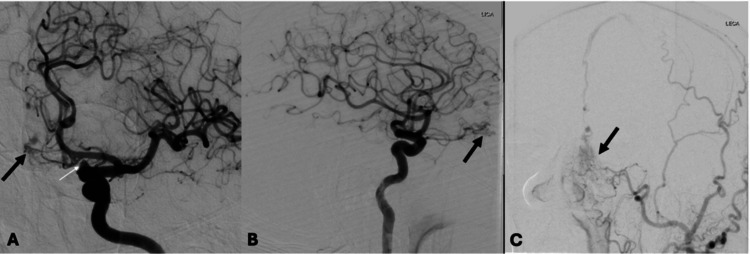
Digital subtraction angiography of left internal carotid artery (ICA) aneurysm and left anterior dural arteriovenous (AV) fistula Cerebral angiogram images demonstrate an unruptured saccular aneurysm (3.7 x 2.8) (white arrow) projecting anterosuperiorly from the left supraclinoid ICA, as well as a left anterior dural AV fistula (black arrow) that is draining into the superior sagittal sinus.

On the morning of his procedure at 8 A.M., the patient demonstrated normal mental status and neurological function. The cerebral angiogram proceeded without complications and with minimal blood loss. The study confirmed the presence of an aneurysm arising from the left supraclinoid internal carotid artery, located beyond the takeoff of the left ophthalmic artery, measuring 3.7 x 2.8 mm (Figure 1A). An additional unexpected finding was identified: a left anterior dural arteriovenous fistula fed by both the left ophthalmic and left internal maxillary arteries, with venous outflow directed to the superior sagittal sinus (Figure 1A-1C).

**Figure 2:**
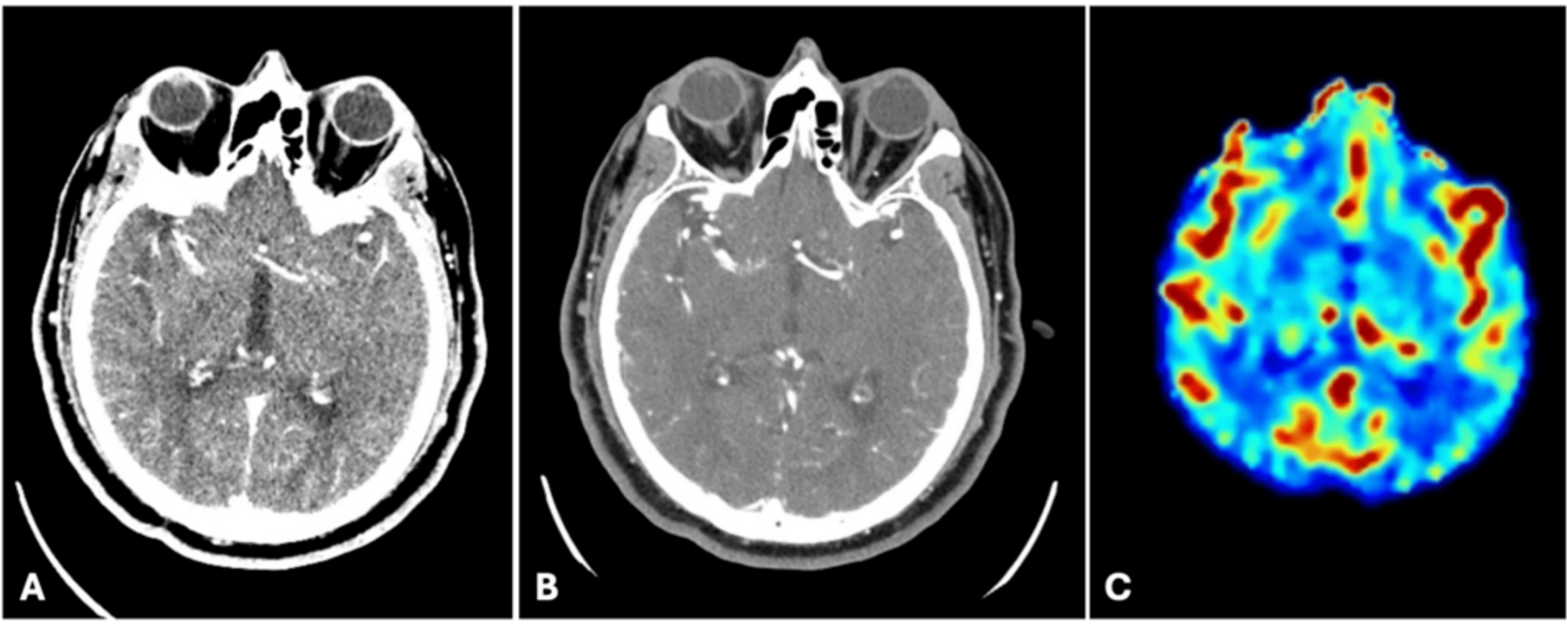
Postoperative CT imaging Non-contrast head CT (A), CT angiogram of the head (B), and CT perfusion (C) demonstrated no acute findings and no perfusion deficits immediately following first sign of neurological deficit.

Post-procedure in the post-anesthesia care unit, the patient woke up with new-onset left-sided sensorimotor deficits. Clinical examination demonstrated markedly diminished strength (2/5) in both the left upper and lower extremities, accompanied by reduced sensation throughout the left side. These acute neurological changes prompted immediate neurology consultation and stroke code activation. Thrombolytic therapy with Tenecteplase was administered at 11:48 A.M., followed by comprehensive neuroimaging including non-contrast head CT, CT angiography of the head and neck, and CT perfusion imaging (Figure 2A-2C). All imaging studies were negative for acute cerebral infarction or intracranial hemorrhage. The patient was then admitted to the neurological intensive care unit for post-thrombolytic care and continuous neurological surveillance.

The patient's neurological symptoms completely resolved by 4:00 P.M. that same day. Physical examination demonstrated full recovery with bilateral 5/5 strength in all extremities and normal light touch sensation in both upper and lower limbs. He was discharged home the following day in good condition. Throughout the ensuing month, the patient experienced no further stroke-like events, and echocardiographic evaluation revealed normal cardiac function. At three months follow-up, the patient successfully underwent surgical resection of his dural arteriovenous fistula.

## Discussion

Contrast-induced encephalopathy is a known rare complication that follows administration of an iodinated contrast agent in procedures such as cardiovascular interventions for acute coronary syndrome and cerebral angiography [1]. Incidence of transient neurological defects following contrast administration is thought to be around 0.51% per 10,000 patients [3]. Among reported cases of contrast-induced encephalopathy, cerebral and coronary angiography are the most commonly associated procedures [9,10]. In this report, we described a probable instance of contrast-induced encephalopathy that occurred after contrast administration for a cerebral angiogram.

The mechanism of contrast-induced neurotoxicity remains unclear. It is believed that the pathogenesis is likely multifactorial, however, a key mechanism likely involves disruption of the BBB [6,7]. Common risk factors for developing contrast-induced encephalopathy include hypertension, diabetes mellitus, renal impairment, and a history of transient ischemic attacks, all of which are believed to play a role in blood-brain barrier breakdown and compromise [1,2,11]. Notably, the patient in our case study did not have any previously documented risk factors and was otherwise healthy.

Clinical presentations of contrast-induced encephalopathy vary with a wide range of symptoms. A prevailing symptom of contrast-induced encephalopathy in multiple past studies was cortical blindness, along with reduced consciousness and motor deficits [9-11]. In our case, the patient presented with numbness and hemiparesis of the left side. However, there were no reports of blindness or reduced consciousness throughout the event. The patient experienced complete resolution of symptoms within the first day of management, which is consistent with timelines of treatments in past studies. 81.5% to 84.9% of patients are thought to experience complete regression of abnormalities, and clinical course is commonly described to improve within 48 to 72 hours [4,10,11].

Prognosis for contrast-induced encephalopathy is generally favorable, and patients typically recover quickly with supportive treatment including intravenous fluids and close monitoring. Corticosteroids to reduce blood-brain barrier damage and antiseizure medications are also commonly employed [1,10]. Radiographic evidence such as mass effect with edema can frequently be used to help aid in diagnosis of contrast-induced encephalopathy, however, such findings are not always present [8]. In this case, the patient underwent a CT head, CT angiogram head and neck, and CT perfusion imaging, all of which showed no signs of acute abnormalities. Close management and intravenous fluids led to resolution of the patient’s symptoms within the first 24 hours.

## Conclusions

Diagnosis of contrast-induced encephalopathy requires early recognition and awareness. The wide presentation of symptoms and variable risk factors lead to challenges in diagnosis. Fortunately, with supportive management, it generally has a favorable outcome. Contrast-induced encephalopathy is often a diagnosis of exclusion, and it should be considered in the differential diagnosis of stroke following procedures involving contrast administration.
